# Number of Positive Lymph Nodes Combined with the Logarithmic Ratio of Positive Lymph Nodes predicts Survival in Patients with Non-Metastatic Larynx Squamous Cell Carcinoma

**DOI:** 10.7150/jca.67348

**Published:** 2022-03-14

**Authors:** Qiyue Wang, Zhuo Tan, Chuanming Zheng, Jiafeng Wang, Guowan Zheng, Ping Huang, Yiwen Zhang, Minghua Ge

**Affiliations:** 1 Jinzhou Medical University, Department of postgraduate education, Jinzhou, Liaoning Province, China.; 2 ENT-Head and Neck Surgery Center, Department of Head and Neck Surgery, Zhejiang Provincial People's Hospital, Affiliated People's Hospital, Hangzhou Medical College, Hangzhou, Zhejiang, China.; 3 Clinical Pharmacy Center, Department of Pharmacy, Zhejiang Provincial People's Hospital, Affiliated People's Hospital, Hangzhou Medical College, Hangzhou, Zhejiang, China.; 4 Key Laboratory of Endocrine Gland Diseases of Zhejiang Province, Hangzhou, Zhejiang, China.

**Keywords:** SEER database, Nomogram, Larynx squamous cell carcinoma, Number of lymph nodes to positive lymph nodes, Logarithmic ratio of positive lymph nodes, Number of positive lymph nodes

## Abstract

**Background:** Logarithmic ratio of positive lymph nodes (LODDS), number of positive lymph nodes (NPLN), and number of lymph nodes to positive lymph nodes (pLNR) are three lymph node classifications; however, their function in prognosis is unclear.

**Purpose:** To establish and validate an optimal nomogram according to the comparison among the 7^th^ TNM stage of American Joint Committee on Cancer (AJCC) and the three lymph node classifications.

**Methods:** A total of 881 patients from the Surveillance, Epidemiology and End Result (SEER database) with T_1-4_N_1-3_M_0_ in laryngeal squamous cell carcinoma from 2000 to 2018 were involved. The enrolled patients were allocated randomly into a training cohort and a validation cohort. Univariate cox regression analysis and multivariable cox regression analysis were applied to explore the predictors. The Akaike Information Criterion (AIC) and Harrell's concordance index (C-index) were to measure the predictive value and the accuracy of the prognostic models. Moreover, integrated discrimination improvement (IDI) and net reclassification index (NRI) were also used to assess the predictive abilities to models. According to the optimal model, nomograms were established and compared with 7^th^ TNM stage of AJCC via the decision curve analysis.

**Results:** NPLN, LODDS, and pLNR were three predictors for the overall and cancer-specific survival in the larynx squamous cell carcinoma. According to the AIC, C-index, IDI, and NRI, the model of NPLN combined with LODDS was assumed as the optimal prognostic model. Moreover, the decision curve analysis suggested that the nomogram demonstrated a better predictive performance, compared with the 7^th^ AJCC TNM stage.

**Conclusion:** The proposed nomograms we constructed for larynx squamous cell carcinoma has potential in the prediction of patients after surgery.

## Introduction

Head and neck carcinoma comprise 4% burden each year in the United States, and is the leading cause of cancer-specific death in the world 1. Laryngeal squamous cell carcinoma (LSCC), originating from the laryngeal mucosal epithelium, is a common subtype of head and neck carcinoma that accounted for 184,615 new cases and 99,840 deaths in 2020 2. LSCC is characterized by occult activity, with approximately 60% of the patients in advanced stages at the time of diagnosis 3. Moreover, LSCC is predisposed to cervical lymph node metastasis and local infiltration, which seriously affects the survival rate of patients 4. Current treatment options for early LSCC are improving, which includes surgery, radiotherapy, and chemotherapy 5. In recent years, despite the advances in treatment, LSCC remains a severe health issue.

Most of the latest prognostic studies focus on head and neck squamous cell carcinoma, while the prognostic model of low-grade squamous cell carcinoma is rarely studied. Additionally, the prognosis of patients with LSCC is currently mainly based on the American Joint Commission on Cancer (AJCC) 7^th^ edition Tumor, Lymph node, Metastasis (TNM) staging system, where stage N is determined by the lymphatic area involved 6. However, the AJCC system could not solve the problem of lymph node heterogeneity, which is clinically important. Currently, some scholars have indicated that the number of positive lymph nodes (NPLNs), positive lymph node ratio (pLNR), and log odds of positive lymph nodes (LODDS) can serve as tools for predicting the prognosis of solid cancer 7, 8. However, it remains unclear whether these three lymph node classification systems could provide a better prognosis than the AJCC system for patients with LSCC. LODDS and pLNR are ratio-based nodal evaluation methods, both of which include the NPLNs. LODDS was calculated as follows: LODDS = log (NPLN + 0.50/NDLN - NPLN + 0.50), where NPLN is defined as the number of positive lymph node and NDLN is defined as the number of dissected lymph nodes. pLNR was calculated as follows: pLNR = NPLN / NDLN 9-13.

The Surveillance, Epidemiology, and End Results (SEER) Database, a database of cancer incidence and survival rates in the United States, is complete and comprehensive 14. Our objective was to first compare the predictive effect of the classification of NPLN, LODDS, and pLNR of patients with LSCC on the long-term survival prognosis. Second, we established and validated models to construct selective staging systems to enable the prediction of long-term cancer-specific and overall survival (OS) in these patients using node region information and number of examinations.

## Methods and Materials

### Data selection

We obtained data from the SEER databases, which contains the data for approximately 34.6% population in the United States, with the SEER*Stat Software (the version is 8.3.9.2). The dataset we chose for the statistical research is the 'Incidence SEER 18 Registries Customs Data (with additional treatment fields), and the Nov 2020 Sub (2000-2018)' and the username for downloading the statistics is: 11363-Nov2020 15. The histology and site of the primary tumors were coded from the 3^rd^ edition of the International Classification of Diseases for Oncology (ICD-O-3).

### Cohort classification

A total of 881 patients from the SEER database were selected for our research. The inclusion criteria were as follows: (I) The site and morphology were chosen as “Larynx” according to the TNM 7/CS v0204+Schema in the SEER*Stat software, (II) Patients in the cohort diagnosed with T_1-4_N_1-3_M_0_ from 2000 to 2018 according to the AJCC 7^th^ edition, (III) Patients who were diagnosed with primary LSCC with site codes C32.0, C32.1, C32.2, C32.8, and C32.9, and (IV) Positive pathology confirmation of the histological type as squamous carcinoma (8071/3, 8072/3, 8074/3, 8082/3, and 8083/3) based on ICD-O-3 His/Behave. Patients were excluded for the following reasons: (I) Missing or unknown clinical patients information, (II) Survival time of patients was equal to 0 months, (III) Less than one regional lymph node examination or surgery that does not involve lymph node removal, (IV) Patients receiving preoperative radiotherapy, (V) Patients with multiple primary cancers, (VI) Lack of information on NDLN, NPLN, and TNM stage and survival outcomes, (VII) The stage according to AJCC were inconformity to T_1-4_N_1-3_M_0_; (VIII) Diagnosis not confirmed by positive histology and death certificates or autopsy. The process of filtering the data is shown in Figure S1.

### Data Processing

After data filtering, additional classification was carried out. Age, NPLN, LODDS, as well as pLNR were considered as continuous variables; the other factors excluding these were considered as categorical variables. Marital status in our study included single, married, discovered, and others. We classified the number of lymph node dissection in patients as 1 to 3, 4 or more, and others. In several clinical studies, the correlation is not linear between the continuous variables and the outcomes; thus, the clinical application of the continuous variables is challenging. We used X-tile software to identify the best threshold for the survival data 16. The software performs statistical tests on different values as cutoff values, and the optimal cutoff value is identified as the smallest *p*-value result. Five continuous variables (Age, Tumor size, NPLN, LODDS, and pLNR) were trichotomized via the X-tile software. For the OS cohort, age was categorized into 23-64, 65-70, and 71 years and more, while in the cancer-specific survival (CSS) cohort age was categorized into 23-63, 64-70, and 71 years and more. Tumor size was divided into low size (1-21 mm), middle size (22-53 mm), and high size (54-110 mm) in the OS cohort, while in the CSS cohort, tumor size was divided into low size (1 -30 mm), middle size (31-48 mm) and high size (49-110 mm). LODDS was grouped into low LODDS (-2.26 to -1.36), middle LODDS (-1.35 to -0.93), and high LODDS (-0.93 to 0.70) in the OS cohort, while in the CSS cohort, LODDS was grouped into low LODDS (-2.26 to -1.35), middle LODDS (-1.34 to -0.87), high LODDS (-0.86 to 0.7). pLNR was divided into low pLNR(0 to 0.04), middle pLNR (0.05 to 0.09), high pLNR (-0.93 to 0.70) in the OS cohort; while pLNR was divided into low pLNR (0 to 0.04), middle pLNR (0.05 to 0.11), and high pLNR (0.34 to 1) in the CSS cohort. NPLN was divided into low NPLN (0-1), middle NPLN (2-5), high NPLN (6-43) in both the OS and CSS cohort.

In our study, we chose OS and CSS as the primary endpoints. OS was defined as the time from the beginning to death from any causes. CSS was defined as the time until cancer results in the death of the individual. The prognosis and follow-up information from the SEER database are updated regularly, with the latest data published in 2020.

### Establishment of a prognostic model

To distinct the prognostic model, continuous variables were converted to rank variables or categorical variables and presented as counts and proportions. The enrolled patients were randomly allocated into a training and a validation cohort by 7:3 proportion, analyzing the clinical prognostic information for those two groups. Univariate cox regression analysis was applied to define the potential prognostic factors in the training cohort 17. After excluding the non-statistically significant prognostic factors, the remaining statistical significance factors (*p* < 0.1) were included in the multivariate cox regression analysis. 95% confidence intervals (CI) and Hazard ratio (HR) were also presented. Secondly, we examined the correlation between the model of NPLN, pLNR, and LODDS of the overall status, training cohort, and validation cohort. Thirdly, we included NPLN, pLNR, LODDS, pLNR + NPLN, and LODDS + NPLN into five disparate multivariate Cox regression models. The performance prediction of these five models was evaluated by the statistical model fit, discriminatory ability, and accuracy. The Akaike Information Criterion (AIC) was to evaluate the fit for the statistical model 18. Harrell's concordance index (C-index) was applied to measure the accuracy and discriminatory ability for the predictive models 19. The net reclassification index (NRI) and integrated discrimination improvement (IDI) index were used to assess improvement in the predictive models.

### Construction of the nomograms

The multivariate cox regression models of OS and CSS were converted to nomograms with optimized performance prediction, which were constructed using the R software 20. Moreover, 1-, 3-, and 5-year OS and CSS were calibrated via calibration curves to compare the nomogram-predicted survival with the actual survival 21. Decision curve analysis (DCA) was used for the comparison among the TNM stage and the nomogram 22.

### Statistical Analysis

We used R (version 4.1.1) to perform all the statistical tests. Kaplan-Meier analysis was used to calculate the survival time in both OS and CSS, and the log-rank test was used to analyze the differences in the survival curves. Spearman's correlation coefficients were used to confirm collinearity among the variables. The correlation coefficient between the two independent variables considered as absence of multicollinearity was less than 0.7 23. All the tests deemed as statistically significant were two-sided, and a *p* value < 0.05 was set.

## Results

### Clinicopathological Characteristics

The SEER program currently collects and publishes the morbidity, treatment information, and survival data from more than 26% of the population-based cancer registries in the United States. Overall, a total of 59,526 patients diagnosed with LSCC between January 2010 and December 2015 were enrolled in the research. After adoption of the screening criteria, 881 patients were incorporated in the final study cohort, of which 615 were assigned to the training cohort and 266 to the validation cohort. The basic characteristics of the patients in both the training and validation cohorts are displayed in Table 1. The median age at diagnosis was 61 years.

### Survival analysis

**Figure 1:**
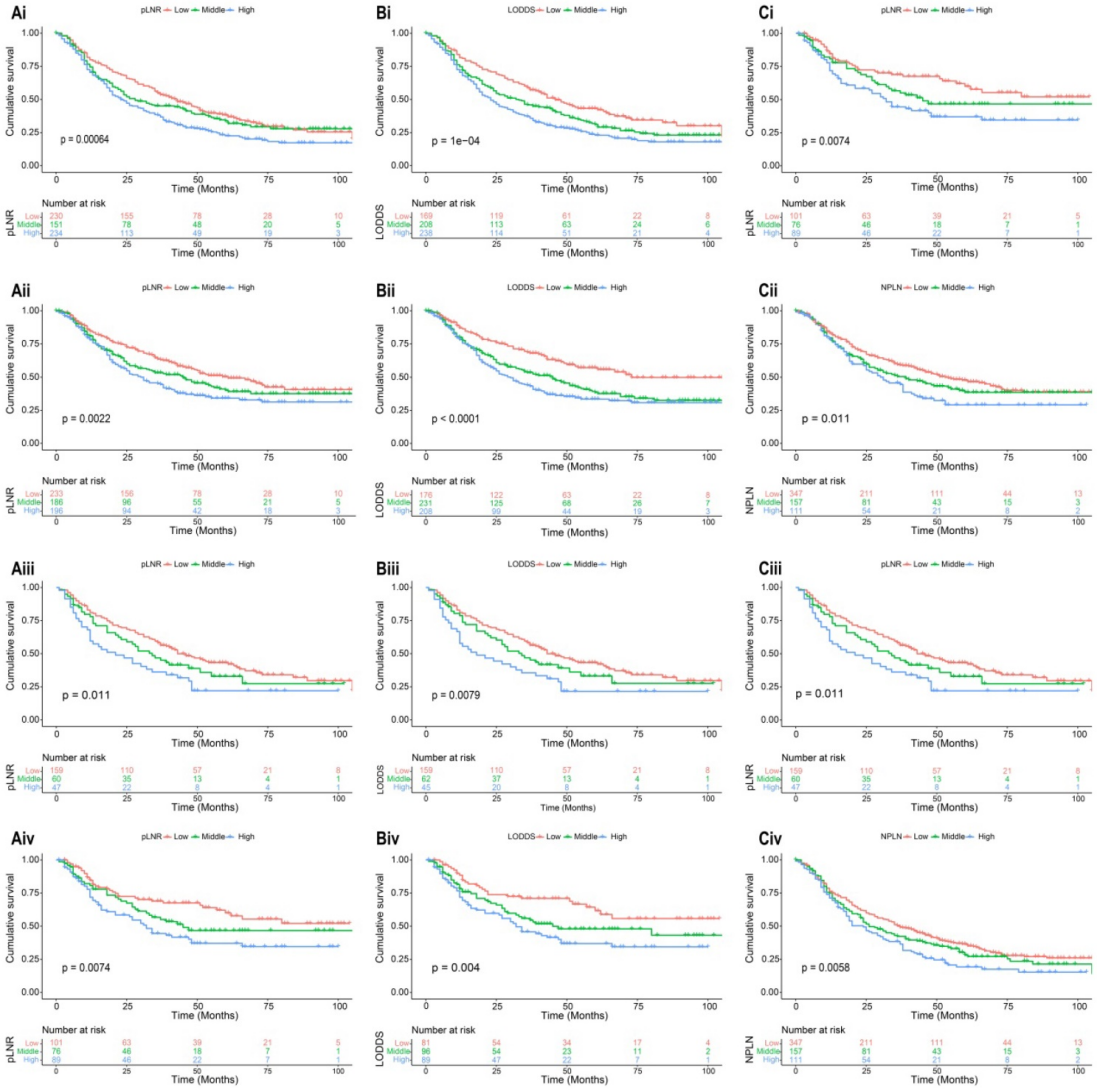
Kaplan-Meier analysis of OS and CSS for patients with high, middle, and low pLNR in training cohort [(Ai), OS; (Aii), CSS] and validation cohort [(Aiii), OS; (Aiv), CSS]; for patients with high, middle, and low LODDS in training cohort [(Bi), OS; (Bii), CSS] and validation cohort [(Biii), OS; (Biv), CSS]; for patients with high, middle, and low NPLN in training cohort [(Ci), OS; (Cii), CSS] and validation cohort [(Ciii), OS; (Civ), CSS].

**Figure 2:**
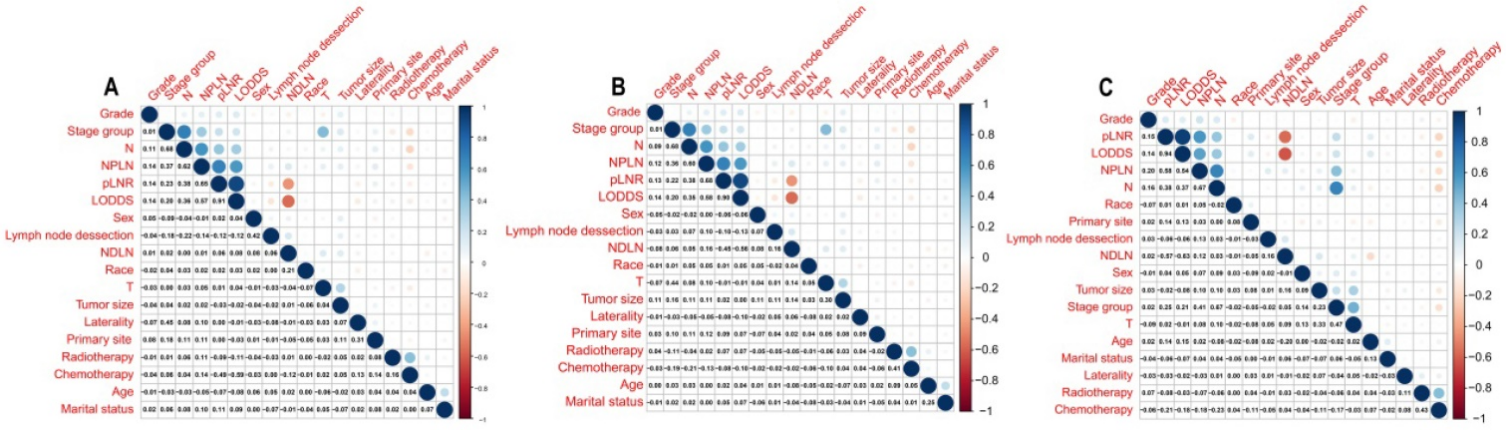
Correlation among variables in overall cohort (**A**), training cohort (**B**) and validation cohort (**C**). pLNR shows the significance correlation with LODDS in three cohorts.

The median survival-months' time on the whole was 10 months upon calculating the time from patient admission to the cutoff date for analysis 24. As displayed in Figure 1, patients with a high value of pLNR, LODDS, and NPLN have a significant correlation with lower OS and CSS rates that the log-rank test is *p* < 0.05. However, there was a significant correlation between the pLNR and LODDS (Spearman correlation is 0.9); moreover, there was no significant association among the NPLN and the other variables in the three datasets, which includes the overall set, the training cohort as well as the validation cohort (Figure 2). Thus, we constructed five models of pLNR, LODDS, NPLN, pLNR + NPLN, and LODDS + NPLN to investigate the predictive potential.

**Figure 3:**
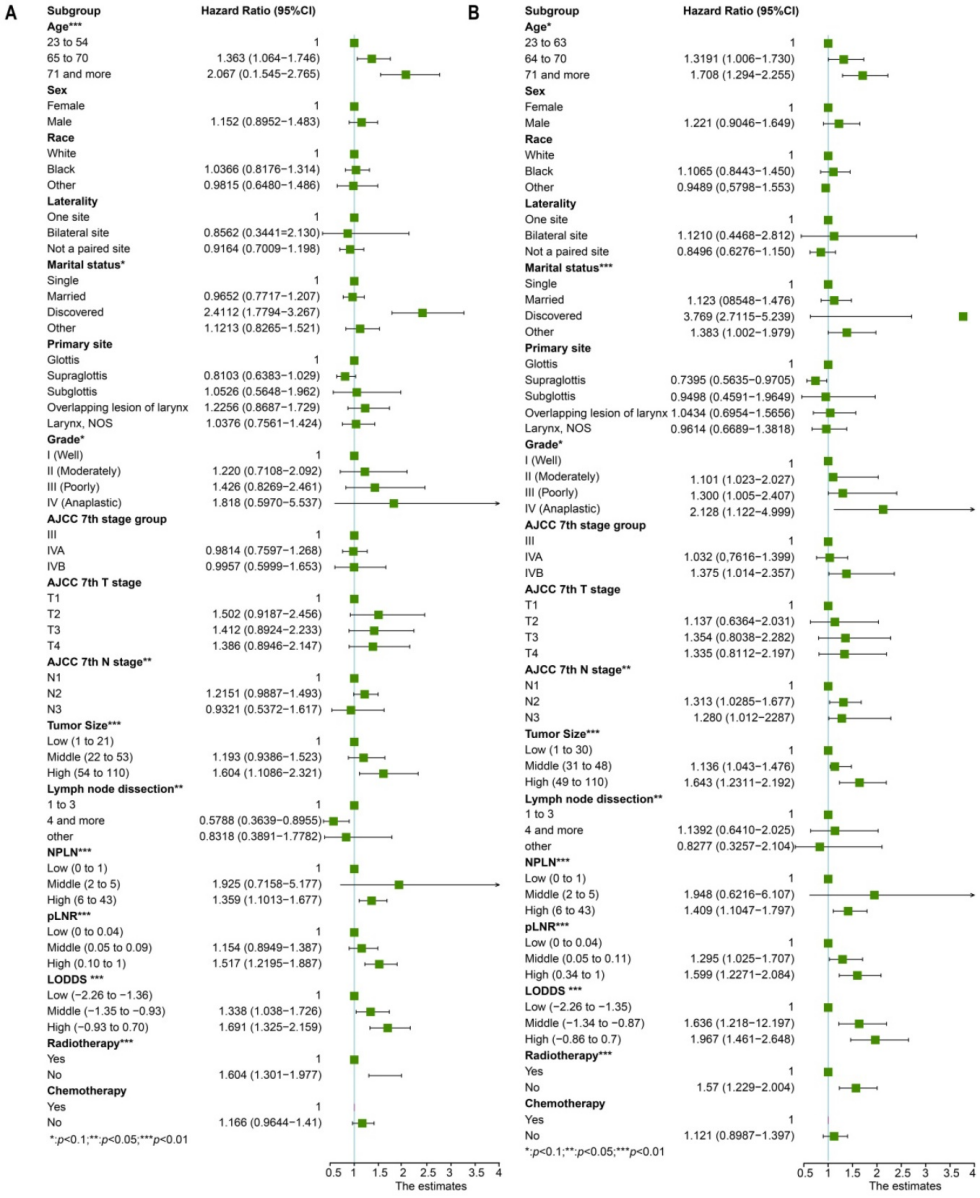
The forest plot for univariate cox regression analysis in OS (**A**) and CSS (**B**). *Means: *p* < 0.1; ** Means: *p* < 0.05; ***Means: *p* < 0.01.

**Figure 4:**
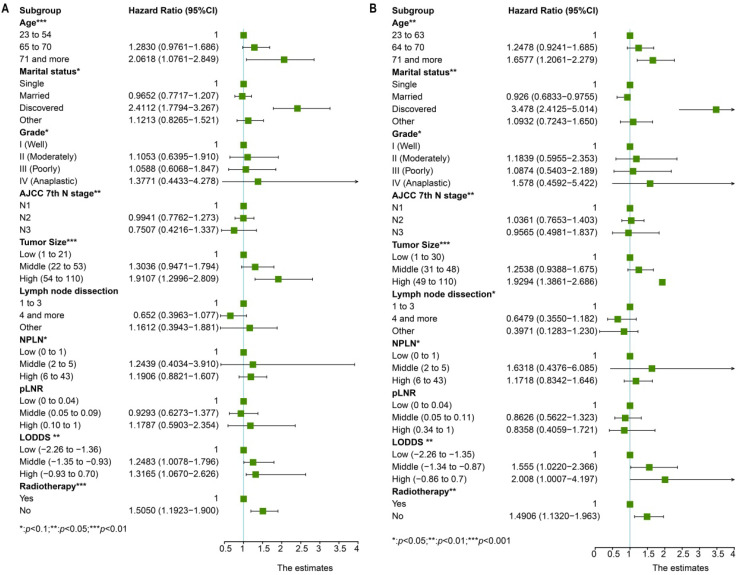
Multivariate cox regression analysis and forest plot of prognostic predictors for OS (**A**) and CSS (**B**). *Means: *p* < 0.1; ** Means: *p* < 0.05; ***Means: *p* < 0.01.

Univariate cox regression analysis included the following factors: age, sex, race, marital status, grade, laterality, primary site, AJCC 7^th^ T stage, AJCC 7^th^ N stage, AJCC 7^th^ M stage, tumor size, lymph node dissection, pLNR, NPLN, LODDS, radiotherapy condition, and chemotherapy condition. The prognostic factors involved in the multivariate cox regression analysis were the factors with significant differences (*p* < 0.1) in the univariate cox regression analysis for both OS and CSS. Additionally, we incorporated five models, NPLN, pLNR, LODDS, pLNR + NPLN, and LODDS + NPLN, into the multivariate cox regression models. The cox regression analyses revealed that sex, primary site, race, chemotherapy, and T classification have no significance on both the OS and CSS. The results for the univariate cox regression analysis of both the OS and CSS are displayed in Figure 3, and those of the multivariate cox regression analysis are shown in Figure 4.

### Comparison of the predictive performance among the models

We tested the AIC among the five models and found that LODDS + NPLN demonstrated lower AIC than the other four models. The trend was similar to the C-index, which showed that the model of LODDS with NPLN displayed higher accuracy than the other four models (Table 2). In other words, the model of LODDS + NPLN exhibited superior predictive potential than the other four models. The comparison between LODDS with NPLN and the other models using integrated discrimination improvement (IDI) and net reclassification index (NRI) are displayed in Table 2 25. Both IDI and NRI values were less than 0, illustrating that the model of LODDS combined with NPLN had a better predicting performance.

### Construction and validation of the nomograms

**Figure 5:**
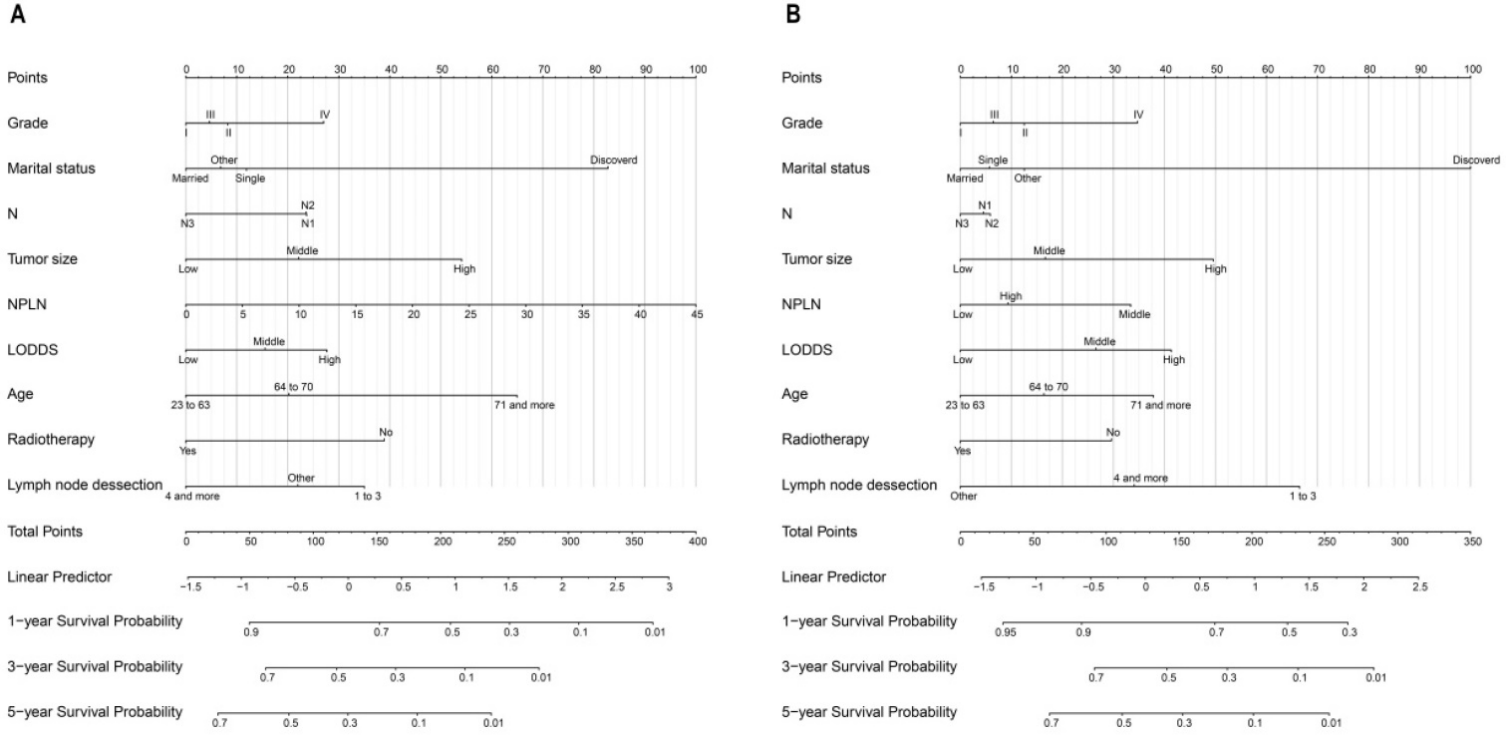
Nomograms for patients to predict 1-year, 3-year and 5-year for OS (**A**) and CSS (**B**).

The nomogram for OS consisted of nine prognostic factors: grade, marital status, N stage, tumor size, NPLN, LODDS, age, radiotherapy situation, and lymph node dissection (Figure 5A). Likewise, the nomogram for CSS has also been established (Figure 5B). The 1-, 3-, and 5-year survival for both OS and CSS could be calculated via those nomograms. As can be seen from the nomogram for OS, NPLN contributed the most to this nomogram, followed by marital status, tumor size, and other variates. While interestingly, for CSS, marital status had the greatest effect, followed by NPLN and lymph node dissection. Each of these variables corresponds to a score on the score sheet. Upon adding up the points, the total score can be obtained, then drawing a line below the total score.

**Figure 6:**
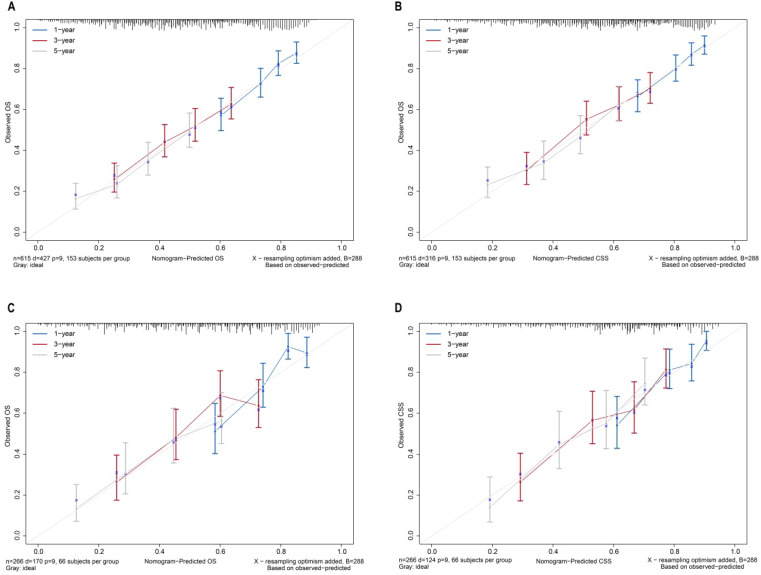
Calibration curves for nomograms in OS, CSS prediction of the training cohort (**A, B**), and validation cohort (**C, D**).

**Figure 7:**
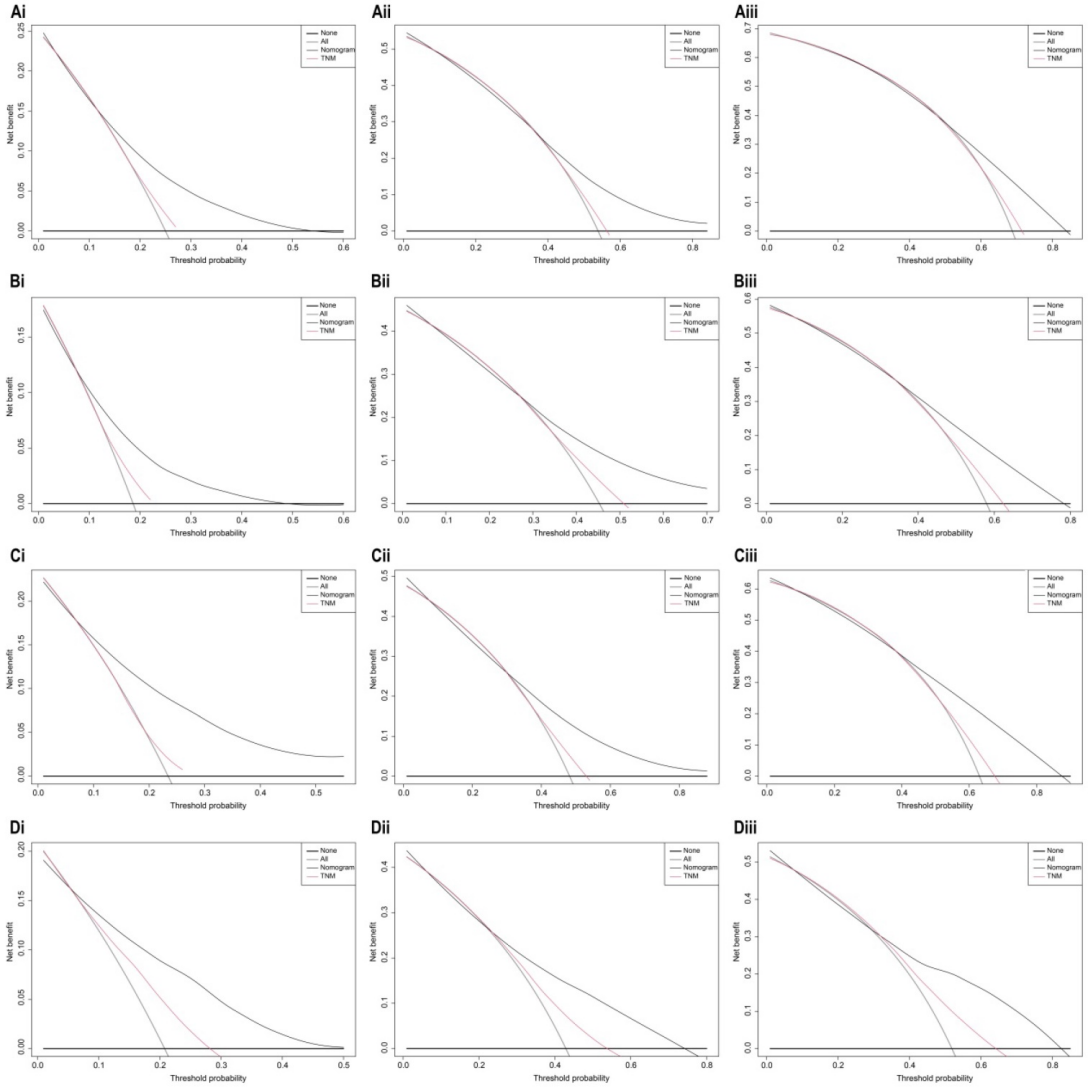
Decision curve analysis of 7^th^ AJCC TNM stage and nomogram for 1-year, 3-year, and 5-year OS (**Ai, Aii, Aiii**), CSS (**Bi, Bii, Biii**) prediction of the training cohort; 1-year, 3-year, and 5-year OS (**Ci, Cii, Ciii**), CSS (**Di, Dii, Diii**) prediction of the validation cohort.

The bootstrap self-sampling method was used to calculate the C-index for OS and CSS in the training cohort, which was 0.654 and 0.671, respectively (Table 2). The predicted calibration curves for the 1-, 3-, and 5-year OS and CSS in the training (Figure 6A, 6B) and validation cohort (Figure 6C, 6D) were similar to the standard curves. The DCA curves of the OS and CSS (Figure 7) demonstrate that LODDS + NPLN has good predictive power for patient prognosis for both the training and validation cohorts.

### Risk stratification

**Figure 8:**
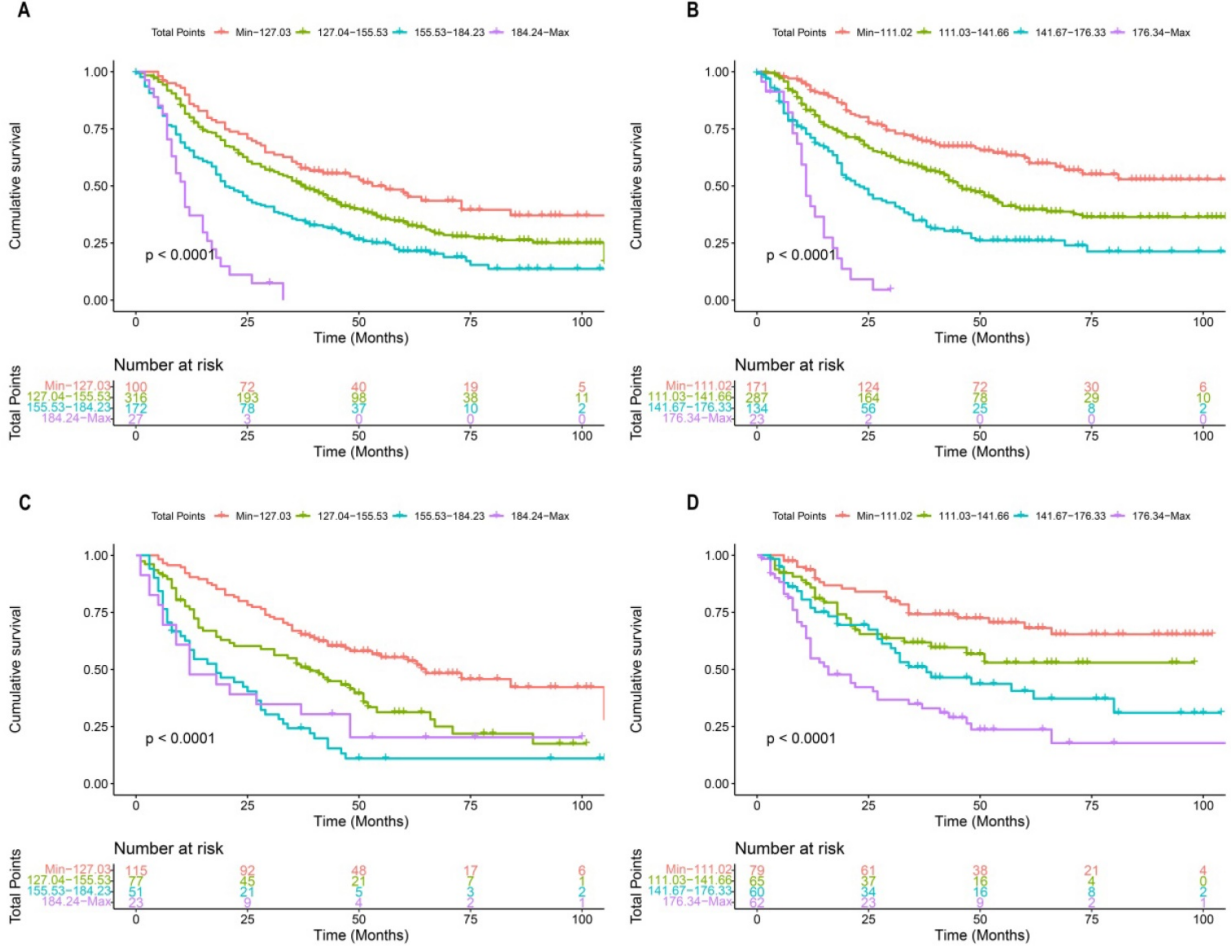
Kaplan-Meier curves of OS and CSS for patients with risk stratification quartering in the training cohort OS (**A**), CSS (**B**) and validation cohort OS (**B**), CSS (**D**).

Each patient in the training cohort and validation cohort were calculated the total score, divided the score into OS (min-127.03, 127.04-155.53, 155.53-184.23, 184.24-max) and CSS (min-111.02, 111.03-141.66, 141.67-176.33, 176.34-max) quartiles represent different results. After stratifying the patients according to quartiles, statistically significant differences were observed between the OS (Figure 8A, 8C) and CSS (Figure 8B, 8D).

## Discussion

Recently, as increasingly many indicators have been identified as prognostic factors for predicting LSCC, the conventional AJCC staging system has gradually lost its power to assess the prognosis. The primary reason is that the AJCC stage is mainly based on the anatomical location of lymph nodes, which could not adequately reflect the disease status. In the last two decades, the LODDS and pLNR have garnered great interest. In the studies by Persiain et al. and Hou X et al., LODDS and pLNR have a better predictive value for the survival time 7, 26. Thus, based on the different lymph node classifications, we constructed different models to figure out the superior predictive potential for prognosis. In our study, we filtrated data from the SEER database, determining the prognostic risk factors based on the cox regression analysis. We concluded that pLNR, NPLN, and LODDS are important factors affecting the survival of patients with T_1-4_N_1-3_M_0_ LSCC and could be applied to predict the prognosis of these patients. The results of the multiple risk factors were visualized, and it was proved that those factors had a significant influence on the prognosis of the patients with LSCC. The value of the C-index, calibration curve, as well as the DCA curve also demonstrated that the nomogram we constructed has good clinical prediction ability. In addition, risk stratification demonstrated the feasibility of the nomogram in patients with clinically different cancer stages.

To treat patients with LSCC effectively and accurately, prognostic factors that may influence survival are to be considered. Nowadays, AJCC stage of carcinoma of the larynx is mainly based on the anatomical location of the lymph nodes, without considering the number and ratio of the positive lymph nodes. However, the pLNR, LODDS, and NPLN were demonstrated as independent prognostic predictors previously, during the release of AJCC 7^th^ guidelines. We demonstrated that the NPLN, LODDS, and pLNR were independent predictors; pLNR has significant correlation, and LODDS combined with NPLN has a pivotal effect on the prognosis. Thus, future studies should focus on the prognostic value of LODDS and NPLN.

To our knowledge, this is the first new model that combines LODDS and NPLN to predict long-term survival in patients with LSCC. Previous research has focused on the comparison of the lymph node ratio 27; we creatively incorporated the LODDS and NPLN in a prognosis model, which strengthened the TNM staging and utilized the valuable pathological evidence for surgery. We believe when compared with the other four models, LODDS + NPLN shows the most significant predictive potential, that is, the higher the value of LODDS with NPLN, the lower the value of OS and CSS. Future treatment strategies need to be studied for better patient survival.

We demonstrated that the patients who received radiotherapy were correlated with a better CSS or OS compared with those who were not treated with radiotherapy. Our results are consistent with the actual condition of the clinical treatment. Owing to sensitivity to chemotherapy drugs, many patients opt for radiotherapy as adjuvant therapy in clinical practice 1. Another interesting finding in our study was that the T stage had no influence on both the OS and CSS of patients. We believe this is because the head and neck squamous cell carcinoma is characterized by a higher rate of lymph node metastasis; therefore, the degree of tumor invasion may have little impact on the prognosis of the disease 28-30. Surprisingly, we found that marital status plays an important role in the disease prognosis, with married patients having higher cancer survival rates than unmarried patients 31. For other cancers, such as lung, pancreatic, and breast cancers, married patients also had a better prognosis than unmarried patients 31, 32. However, the effects of the correlation between long-term survival and other subtypes of marital status require further research.

Finally, based on the LODDS + NPLN model, two kinds of convincing nomograms with high C-indexes were established, which were verified by bootstrap technology internally and calibration curve externally. The prediction of the OS and CSS with the column line shows a good calibration diagram. The nomogram consists of a number of readily available prognostic factors that can help physicians assess the risk of death, counsel patients, and make decisions. Therefore, patients with poor survival who might need more aggressive treatment, which includes both chemotherapy and radiation, can be identified based on the nomogram.

Our study also has certain limitations. First, the SEER database lacks several potential prognostic factors, for instance, the specific chemotherapy regimen, tyrosine kinase inhibitor therapy, immune checkpoint inhibitor therapy, etc. Second, since this was a population study, we could not use a unified counting method, which could lead to the underestimation of lymph nodes when they adhered to each other or were difficult to separate from the anatomical tissue, and the overestimation of lymph nodes in the case of lymphatics. Third, no relapse-free survival was recorded in the SEER database. Finally, only the patients from the United States are included in the SEER database currently, which, while rich, may not be an ideal representation of the patients in other regions.

## Conclusions

We confirmed that LODDS combined with NPLN was superior to pLNR, LODDS, NPLN, and pLNR combined with NPLN in predicting the survival prognosis of patients undergoing laryngeal surgery. A dynamic nomograph including TNM was constructed, supplemented by LODDS and NPLN to assess both the OS and CSS. These nomograms could help doctors provide efficient and more personalized treatment for patients with laryngeal cancer.

## Supplementary Material

Supplementary figure.

## Figures and Tables

**Table 1:** Baseline characteristics of training cohort and external validation cohort

	Total	Training cohort		Validation cohort		*p*-value
		N	%	N	%	
Numbers of patients	881	615	69.81	266	30.19	
**Age (years)**						
median (IQR)		61 (54-68)		60 (55-68)		0.9182
**Sex**						
Female	160	102	63.75	58	36.25	0.1277
Male	721	513	71.15	208	28.85	
**Race**						
Black	663	464	69.98	199	30.02	1
White	173	120	69.36	53	30.64	
Other	45	31	68.89	14	31.11	
**Laterality**						
One site	125	93	74.40	32	25.60	0.3549
Bilateral site	14	11	78.57	3	21.43	
Not a paired site	742	511	68.87	231	31.13	
**Marital status**						
Single	319	227	71.16	92	28.84	0.9230
Married	362	249	68.78	113	31.22	
Discovered	80	56	70.00	24	30.00	
Others	120	83	69.17	37	30.83	
**Primary site**						
Glottis	206	149	72.33	57	27.67	0.8144
Supraglottis	454	318	70.04	136	29.96	
Subglottis	24	16	66.67	8	33.33	
Overlapping lesion of larynx	82	54	65.85	28	34.15	
Larynx, NOS	115	78	67.83	37	32.17	
**Grade**						
I (Well)	39	30	76.92	9	23.08	0.5638
II (Moderately)	473	334	70.61	139	29.39	
III (Poorly)	352	237	67.33	115	32.67	
IV (Anaplastic)	7	4	57.14	3	42.86	
**AJCC 7^th^ stage group**						
III	151	101	66.89	50	33.11	0.5354
IVA	693	486	70.13	207	29.87	
IVB	37	28	75.68	9	24.32	
**AJCC 7^th^ T stage**						
T1	50	33	66.00	17	34.00	0.9354
T2	108	76	70.37	32	29.63	
T3	239	166	69.46	73	30.54	
T4	484	340	70.25	144	29.75	
**AJCC 7^th^ N stage**						
N1	298	205	68.79	93	31.21	0.5574
N2	555	388	69.91	167	30.09	
N3	28	22	78.57	6	21.43	
**Lymph node dissection**						
1 to 3	33	24	72.73	9	27.27	0.968
4 and more	831	579	69.68	252	30.32	
other	17	12	70.59	5	29.41	
**NDLN**						
0-20	183	132	72.13	51	27.87	0.6587
21-40	236	168	71.19	68	28.81	
41 and more	468	321	68.59	147	31.41	
**NPLN**						0.1672
median (IQR)		2 (1 ~ 4)		2 (1 ~ 4)		
**Tumor Size (mm)**						0.1025
median (IQR)		36 (28 ~ 47)		35 (28 ~ 45)		
**pLNR**						0.3638
median (IQR)		0.063 (0.030 ~ 0.149)		0.058 (0.027 ~ 0.131)		
**LODDS**						0.2509
median (IQR)		-1.086 (-1.386~-0.699)		-1.140 (-1.1424 ~ -0.7685)		
**Radiotherapy**						
Yes	658	453	68.84	205	31.16	0.3251
No	223	162	72.65	61	27.35	
**Chemotherapy**						
Yes	403	289	71.71	114	28.29	1
No	478	326	68.20	152	31.80	

**Table 2:** Comparison of prognostic models in the training cohort

Model	AIC	C-index	NRI (95%CI)	*p* value	IDI (95%CI)	*p* value
**Cancer-specific survival**						
NPLN	3670.673	0.64	-0.001 (-0.034 to 0.019)	0.32	-0.003 ( -0.006 to -0.001)	0.002
LODDS	3667.561	0.645	-0.000 (-0.054 to 0.029)	0.884	-0.001 (-0.002 to 0.014)	0.580
pLNR	3670.350	0.641	-0.002 (-0.004 to -0.000)	< 0.001	-0.006 (-0.008 to -0.004)	< 0.001
pLNR + NPLN	3672.063	0.642	-0.006 (-0.010 to -0.002)	<0.001	-0.101 (-0.142 to -0.0879)	< 0.001
LODDS + NPLN	3665.249	0.671	Reference		Reference	
**Overall survival**						
NPLN	4929.827	0.635	-0.008 (-0.013 to -0.005)	< 0.001	-0.006 (-0.010 to -0.002)	< 0.001
LODDS	4929.366	0.633	-0.067 (-0.131 to 0.039)	0.266	-0.001 (-0.002 to -0.000)	0.023
pLNR	2932.002	0.628	-0.094 (-0.125 to -0.068)	< 0.001	-0.004 (-0.006 to -0.003)	< 0.001
pLNR + NPLN	4928.131	0.649	-0.088 (-0.136 to -0.066)	< 0.001	-0.002 (-0.003 to -0.000)	< 0.001
LODDS + NPLN	4925.764	0.654	Reference		Reference	

## Abbreviations

LSCC: laryngeal squamous cell carcinoma
AJCC: American Joint Commission on Cancer
TNM: tumor, lymph node, metastasis
LODDS: logarithmic ratio of positive lymph nodes
NPLN: number of positive lymph nodes
pLNR: number of lymph nodes to positive lymph nodes
SEER: Surveillance, Epidemiology, and End Result
ICD-O-3: International Classification of Disease for Oncology
OS: overall survival
CSS: cancer-specific survival
AIC: Akaike Information Criterion
C-index: Harrell's concordance index
IDI: integrated discrimination improvement
NRI: net reclassification index

## References

[1] Cramer JD, Burtness B, Le QT, Ferris RL (2019). The changing therapeutic landscape of head and neck cancer. Nat Rev Clin Oncol.

[2] Yan K, Agrawal N, Gooi Z (2018). Head and Neck Masses. Med Clin North Am.

[3] Bray F, Ferlay J, Soerjomataram I, Siegel RL, Torre LA, Jemal A (2018). Global cancer statistics 2018: GLOBOCAN estimates of incidence and mortality worldwide for 36 cancers in 185 countries. CA Cancer J Clin.

[4] Bayir O, Toptas G, Saylam G, Izgi TC, Han U, Keseroglu K Occult lymph node metastasis in patients with laryngeal cancer and relevant predicting factors: a single-center experience. Tumori. 2021: 3008916211026977.

[5] Steuer CE, El-Deiry M, Parks JR, Higgins KA, Saba NF (2017). An update on larynx cancer. CA Cancer J Clin.

[6] Wu Y, Zhang Y, Zheng X, Dai F, Lu Y, Dai L (2020). Circular RNA circCORO1C promotes laryngeal squamous cell carcinoma progression by modulating the let-7c-5p/PBX3 axis. Mol Cancer.

[7] Hou X, Wei JC, Xu Y, Luo RZ, Fu JH, Zhang LJ (2013). The positive lymph node ratio predicts long-term survival in patients with operable thoracic esophageal squamous cell carcinoma in China. Ann Surg Oncol.

[8] Ramacciato G, Nigri G, Petrucciani N, Pinna AD, Ravaioli M, Jovine E (2017). Prognostic role of nodal ratio, LODDS, pN in patients with pancreatic cancer with venous involvement. BMC Surg.

[9] Horn S, Ozsahin M, Lefebvre JL, Horiot JC, Lartigau E, Association of R, et al (2012). Larynx preservation: what is the standard treatment?. Crit Rev Oncol Hematol.

[10] Pei JP, Zhang CD, Fan YC, Dai DQ (2018). Comparison of Different Lymph Node Staging Systems in Patients With Resectable Colorectal Cancer. Front Oncol.

[11] Liao Y, Yin G, Fan X (2020). The Positive Lymph Node Ratio Predicts Survival in T1-4N1-3M0 Non-Small Cell Lung Cancer: A Nomogram Using the SEER Database. Front Oncol.

[12] Zhang X, Lu L, Shang Y, Liu P, Wei Y, Ma L (2017). The number of positive lymph node is a better predictor of survival than the lymph node metastasis status for pancreatic neuroendocrine neoplasms: A retrospective cohort study. Int J Surg.

[13] Huang B, Ni M, Chen C, Cai G, Cai S (2017). LODDS is superior to lymph node ratio for the prognosis of node-positive rectal cancer patients treated with preoperative radiotherapy. Tumori.

[14] Altekruse SF, Rosenfeld GE, Carrick DM, Pressman EJ, Schully SD, Mechanic LE (2014). SEER cancer registry biospecimen research: yesterday and tomorrow. Cancer Epidemiol Biomarkers Prev.

[15] Doll KM, Rademaker A, Sosa JA (2018). Practical Guide to Surgical Data Sets: Surveillance, Epidemiology, and End Results (SEER) Database. JAMA Surg.

[16] Camp RL, Dolled-Filhart M, Rimm DL (2004). X-tile: a new bio-informatics tool for biomarker assessment and outcome-based cut-point optimization. Clin Cancer Res.

[17] Fisher LD, Lin DY (1999). Time-dependent covariates in the Cox proportional-hazards regression model. Annu Rev Public Health.

[18] Shipley B, Douma JC (2020). Generalized AIC and chi-squared statistics for path models consistent with directed acyclic graphs. Ecology.

[19] Kim S, Schaubel DE, McCullough KP (2018). A C-index for recurrent event data: Application to hospitalizations among dialysis patients. Biometrics.

[20] Iasonos A, Schrag D, Raj GV, Panageas KS (2008). How to build and interpret a nomogram for cancer prognosis. J Clin Oncol.

[21] Austin PC, Harrell FE Jr, van Klaveren D (2020). Graphical calibration curves and the integrated calibration index (ICI) for survival models. Stat Med.

[22] Van Calster B, Wynants L, Verbeek JFM, Verbakel JY, Christodoulou E, Vickers AJ (2018). Reporting and Interpreting Decision Curve Analysis: A Guide for Investigators. Eur Urol.

[23] Waldmann P (2019). On the Use of the Pearson Correlation Coefficient for Model Evaluation in Genome-Wide Prediction. Front Genet.

[24] Liu Q, Li C, Wanga V, Shepherd BE (2018). Covariate-adjusted Spearman's rank correlation with probability-scale residuals. Biometrics.

[25] Demler OV, Pencina MJ, Cook NR, D'Agostino RB Sr (2017). Asymptotic distribution of AUC, NRIs, and IDI based on theory of U-statistics. Stat Med.

[26] Persiani R, Cananzi FC, Biondi A, Paliani G, Tufo A, Ferrara F (2012). Log odds of positive lymph nodes in colon cancer: a meaningful ratio-based lymph node classification system. World J Surg.

[27] Cui J, Wang L, Zhong W, Chen Z, Tan X, Yang H (2020). Development and validation of nomogram to predict risk of survival in patients with laryngeal squamous cell carcinoma. Biosci Rep.

[28] Johnson DE, Burtness B, Leemans CR, Lui VWY, Bauman JE, Grandis JR (2020). Head and neck squamous cell carcinoma. Nat Rev Dis Primers.

[29] Duprez F, Berwouts D, De Neve W, Bonte K, Boterberg T, Deron P (2017). Distant metastases in head and neck cancer. Head Neck.

[30] Sharbel DD, Abkemeier M, Groves MW, Albergotti WG, Byrd JK, Reyes-Gelves C (2021). Occult Metastasis in Laryngeal Squamous Cell Carcinoma: A Systematic Review and Meta-Analysis. Ann Otol Rhinol Laryngol.

[31] Chen Z, Yin K, Zheng D, Gu J, Luo J, Wang S (2020). Marital status independently predicts non-small cell lung cancer survival: a propensity-adjusted SEER database analysis. J Cancer Res Clin Oncol.

[32] Zhai Z, Zhang F, Zheng Y, Zhou L, Tian T, Lin S (2019). Effects of marital status on breast cancer survival by age, race, and hormone receptor status: A population-based Study. Cancer Med.

