# Supplementary figures and images for: Number of Positive Lymph Nodes Combined with the Logarithmic Ratio of Positive Lymph Nodes predicts Survival in Patients with Non-Metastatic Larynx Squamous Cell Carcinoma
Source: J Cancer. 2022 Mar 14;13(6):1773–84. doi: 10.7150/jca.67348 (PMC8990420; doi:10.7150/jca.67348)

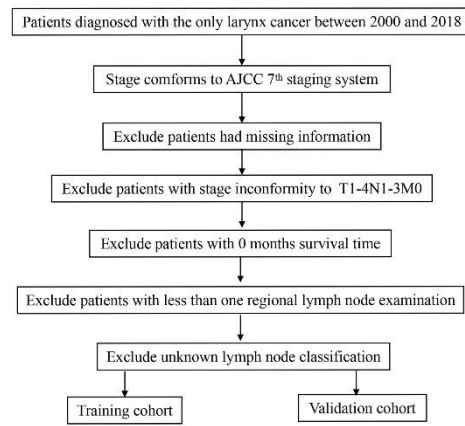

Figure S1. The flow chat of patient selection process.

Supplement: Supplementary file 1 — Supplementary figure. [file jcav13p1773s1.pdf]
